# Recovery from Cycling Exercise: Effects of Carbohydrate and Protein Beverages

**DOI:** 10.3390/nu4070568

**Published:** 2012-06-25

**Authors:** Qingnian Goh, Christopher A. Boop, Nicholas D. Luden, Alexia G. Smith, Christopher J. Womack, Michael J. Saunders

**Affiliations:** 1 Department of Kinesiology, James Madison University, Harrisonburg, VA 22807, USA; Email: qingnian.goh@rockets.utoledo.edu (Q.G.); boopchris@gmail.com (C.A.B.); ludennd@jmu.edu (N.D.L.); womackcx@jmu.edu (C.J.W.); 2 Shaklee Corporation, Pleasanton, CA 94588, USA; Email: asmith@shaklee.com

**Keywords:** athlete performance, muscle damage, sport nutrition, recovery

## Abstract

The effects of different carbohydrate-protein (CHO + Pro) beverages were compared during recovery from cycling exercise. Twelve male cyclists (VO_2peak_: 65 ± 7 mL/kg/min) completed ~1 h of high-intensity intervals (EX1). Immediately and 120 min following EX1, subjects consumed one of three calorically-similar beverages (285–300 kcal) in a cross-over design: carbohydrate-only (CHO; 75 g per beverage), high-carbohydrate/low-protein (HCLP; 45 g CHO, 25 g Pro, 0.5 g fat), or low-carbohydrate/high-protein (LCHP; 8 g CHO, 55 g Pro, 4 g fat). After 4 h of recovery, subjects performed subsequent exercise (EX2; 20 min at 70% VO_2peak_ + 20 km time-trial). Beverages were also consumed following EX2. Blood glucose levels (30 min after beverage ingestion) differed across all treatments (CHO > HCLP > LCHP; *p* < 0.05), and serum insulin was higher following CHO and HCLP ingestion *versus* LCHP. Peak quadriceps force, serum creatine kinase, muscle soreness, and fatigue/energy ratings measured pre- and post-exercise were not different between treatments. EX2 performance was not significantly different between CHO (48.5 ± 1.5 min), HCLP (48.8 ± 2.1 min) and LCHP (50.3 ± 2.7 min). Beverages containing similar caloric content but different proportions of carbohydrate/protein provided similar effects on muscle recovery and subsequent exercise performance in well-trained cyclists.

## 1. Introduction

Endurance athletes commonly perform activities requiring high levels of performance over repeated days, or multiple events on the same day (*i.e.*, track/swim meets, soccer tournaments, tour cycling). Heavy aerobic exercise, particularly with short time periods between sessions, can result in incomplete muscle glycogen replenishment [1], increased indices of muscle disruption and soreness [2,3], and impaired performance in subsequent exercise [4]. Recent studies have suggested that these aspects of muscle recovery may be influenced by the consumption of carbohydrate-protein (CHO + Pro) recovery beverages. For example, CHO + Pro intake following exercise may accelerate muscle glycogen replenishment rates compared to isocaloric CHO beverages consumed at sub-optimal intake rates (<1 g/kg BW/h); and glycogen repletion with CHO + Pro appears similar to optimal carbohydrate doses [5]. Numerous studies have reported that post-exercise CHO + Pro ingestion attenuates changes in markers of post-exercise muscle disruption following exercise, such as creatine kinase (CK) [2,6,7,8], myoglobin [8] and lactate dehydrogenase [6]. Others have reported that CHO + Pro intake is associated with reduced muscle soreness [2,6] and enhanced post-exercise muscle function [7,8] compared to when carbohydrate alone is consumed. However, the effects of CHO + Pro on these markers of recovery remain controversial, as other studies have reported no differences between CHO and CHO + Pro treatments on markers of muscle disruption [9,10,11], muscle soreness [12], or muscle function [9,12]. Perhaps due to the varying effects of CHO + Pro on these factors, consumption of CHO + Pro supplements during recovery has been reported to augment performance during subsequent whole-body exercise in some [4,7,10,13] but not all [3,6,11,14] studies.

The aforementioned evidence suggests a broad potential for CHO + Pro ingestion to improve recovery following heavy endurance exercise. However, we are aware of no studies that have directly compared CHO + Pro beverages with differing levels of protein content, in order to ascertain the influence of varying CHO/Pro proportions. CHO + Pro beverages investigated in the prior studies universally included high carbohydrate content (~50%–80% of total calories), with smaller amounts of protein (~14%–30%). This is presumably due to the importance of carbohydrate for glycogen replenishment; and it is plausible that high protein content in recovery beverages (at the expense of adequate carbohydrate content) would be less effective for recovery from endurance exercise. However, this hypothesis has not been tested. Thus, the purpose of the present study was to compare the effects of multiple recovery beverages with similar caloric loads, but varied proportions of carbohydrate and protein. Specifically, we extended the findings of prior studies by comparing beverages with 0%, 25% and 75% of total calories from protein. These beverages were consumed following a 1-h session of high-intensity aerobic intervals, which was similar to a protocol previously used to detect differing responses between post-exercise recovery beverages [15,16]. We hypothesized that a CHO + Pro beverage containing 25% protein would improve muscle recovery (and subsequent exercise performance) to a greater extent than a beverage with no protein, and that the low carbohydrate content of a CHO + Pro beverage containing 75% protein would be associated with impaired subsequent performance *versus* both of the other beverages (*i.e.*, 75% CHO/25% Pro > 100% CHO > 25% CHO/75% Pro). 

## 2. Experimental Section

### 2.1. Subjects

Endurance-trained male cyclists were recruited to participate in the study, with the objective of having 12–15 subjects complete all testing. Using power calculations from Lipsey [17], a minimum of 12 subjects provided statistical power ≥0.80 (1-β; two tailed) to detect potential treatment effects of 1.2 SD units. For reference, using the protocols described below, a 1 min (2%) difference in subsequent exercise performance (the primary outcome variable) between treatments represented an effect-size of 1.28. These values were estimated using data obtained from reliability testing of the subsequent exercise test (44.5 ± 4.5 min, intra-class correlation between repeated trials of 0.97; additional information in Section 2.7). All potential subjects were experienced cyclists with a minimum of two years experience in cycling/multisport events; without any recent breaks in training (self-reported minimum of at least three days of cycling training per week during the 2 months prior to study onset). In addition, a peak aerobic capacity (VO_2peak_) ≥ 45 mL/kg/min (assessed during preliminary testing) was required for participation in the study. These criteria were applied to obtain a study sample that was well-trained in cycling-specific training (to minimize potential training effects from the study protocols), but were not intended to represent elite cyclists. All fourteen recruited subjects provided written consent to participate in the study, and met the inclusion criteria. One subject dropped out following preliminary testing (citing other time commitments), and another subject was dropped from the study following completion of his first treatment trial (due to an unrelated illness which prevented him from completing subsequent trials). Twelve subjects completed all procedures, and were characterized as follows: age = 25 ± 8 years; height = 178 ± 4 cm; weight = 73 ± 8 kg; VO_2peak_ = 65 ± 7 mL/kg/min; power at VO_2peak_ (W_max_) = 352 ± 34 W (mean ± SD).

### 2.2. Study Design

**Figure 1:**
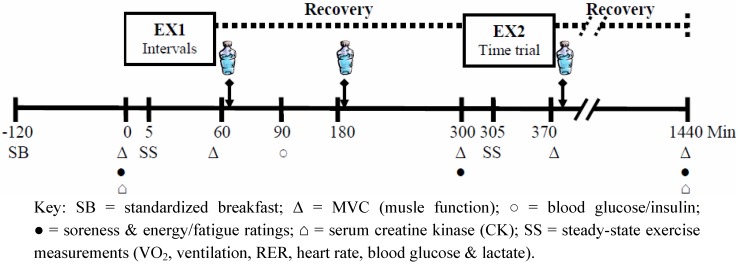
Schematic Representation of the Timeline for each Trial.

Following preliminary testing, subjects completed three experimental trials, with 7–14 days between trials. Each trial began with an exercise session (EX1) consisting of approximately 1 h of high-intensity cycling intervals on an electrically braked cycle ergometer (Velotron, RacerMate, Inc., Seattle, WA, USA). This was followed by a 4-h period of passive recovery, during which time a recovery beverage was consumed on two occasions (immediately and 120 min post-exercise). After the recovery period, a subsequent bout of exercise (EX2, ~70 min of heavy cycling) was performed, followed by a third recovery beverage. Protocols (described in detail below) were identical across all trials, with the exception that a different treatment beverage was utilized in each trial. Treatments included a carbohydrate-only beverage (CHO), a high carbohydrate/low protein beverage (HCLP), and a low-carbohydrate-high-protein beverage (LCHP), which are described below. A double-blind, crossover design with randomly counterbalanced treatment order was employed. Figure 1 provides a schematic illustration of the experimental trials, and dependent measurements obtained during the trials. All protocols were approved by the institutional review board of James Madison University. 

### 2.3. Preliminary Testing

Prior to the commencement of experimental trials, subjects completed two preliminary testing sessions. In the first session, height and weight were recorded, and subjects completed a graded exercise test to determine VO_2peak_ and W_max_, as described previously [18]. Briefly, the test began with subjects riding the same electrically braked cycle ergometer described above at self-selected pace between 100 and 150 W. Power output was subsequently increased by 25 W every 2 min until volitional fatigue. VO_2peak_ and W_max_ were obtained from the test to determine if subjects met entry criteria for the study, and to establish intensities for subsequent exercise testing. 

During the second preliminary testing session, subjects performed a familiarization trial. The purposes of the familiarization trial were to (a) determine if the prescribed exercise intensities were appropriate for each subject (workloads were modified, as necessary, if subjects completed <45 min or >90 min of total work in the initial exercise session of the familiarization trial); and (b) acclimate subjects to the exercise protocols, with the goal of minimizing any possible order-effects in the subsequent treatment trials. Blood samples were not obtained, and none of the 24 h post-exercise measurements were conducted during the familiarization trial. Otherwise, the familiarization mimicked the protocols described for the experimental trials. All subjects received the CHO beverage during the familiarization trial.

### 2.4. Experimental Trials

Subjects completed a diet and exercise log for 48 h prior to each experimental trial (EX1) and throughout each trial period. Participants were instructed to maintain consistent dietary and exercise habits between trials, and to perform no heavy exercise for two days prior to each trial. Subjects consumed their final self-selected meal ≥12 h prior to the start of the exercise protocol (*i.e.*, dinner the evening prior to testing). On the morning of the trial, subjects consumed a standardized breakfast 2 h prior to EX1. The meal was provided by the researchers, and consisted of 495–510 kcal (90–98 g carbohydrate, 8–12 g protein and 4.5–7.5 g fat). Subjects chose from one of 4 meal choices within these macronutrient parameters, and the meal chosen by each subject was repeated across all trials. No nutrients were consumed between EX1 and EX2, or for 2 h following EX2, other than the treatment beverages provided by the researchers. Subjects resumed their normal dietary habits from 2 h following EX2 through the remainder of the data collection period. Total caloric intake, and carbohydrate, protein, and fat content were calculated (using Diet Analysis Plus 8.0™ software) for 24 h prior to each trial (EX1; including standard breakfast) and 24 h following each EX1 (*i.e.*, until 24 h post-exercise testing was completed). No differences were observed in macronutrient levels between trials at either time-point (data not reported). 

### 2.5. Initial Exercise Session (EX1)

Following a warm-up of 10 min at 60% W_max_, subjects completed a series of high-intensity intervals, consisting of 2 min at 95% W_max_ followed by 2 min at 50% W_max_. Subjects rode at a self-selected cadence (usually starting at 95–105 rpm), and were instructed to maintain their cadence above 70 rpm throughout the intervals. If cadence dropped below this level, subjects were verbally encouraged to increase their cadence above 70 rpm. Subjects repeated the high-intensity intervals until they were unable to maintain a cadence of 70 rpm (>10 s) during the intervals at 95% W_max_. 

Workloads for the high-intensity intervals were subsequently decreased to 85% W_max_ until 70 rpm could not be maintained. Workload for the high-intensity intervals was further decreased to 75% W_max_. The test was terminated when subjects failed to maintain a cadence of 70 rpm at this intensity. Recent investigations by Karp [15] and Thomas [16] utilized similar protocols to examine post-exercise nutritional treatments. Based on pilot testing data, we modified the workloads slightly (*i.e.*, 95% W_max_ *versus* 90% W_max_) in order to achieve similar total test duration (~1 h) to these prior investigations. Subjects consumed no nutrients during EX1, but were permitted to consume water *ad libitum* throughout exercise. 

### 2.6. Recovery Period

Following EX1 there was a 4 h period of recovery. Subjects consumed a recovery beverage immediately following this trial and again 120 min post-exercise (described below). 

### 2.7. Subsequent Exercise Performance (EX2)

EX2 was conducted on the same computerized cycle ergometer used for preliminary testing and EX1. The initial 20 min of exercise was performed at 60% W_max_. This portion of the trial was conducted at the same absolute exercise intensity as the initial segment of EX1, allowing a direct comparison of metabolic responses (*i.e.*, VO_2_, RER, blood glucose, lactate, *etc.*) between the two exercise sessions. This segment was immediately followed by a simulated 20 km time trial, which concluded with a challenging 5 km uphill segment (~5% grade). All trials were performed at ambient room temperature (70–72 °F). An oscillating fan was placed two meters from the ergometer, and set at a consistent speed during the trials. Water was provided *ad libitum* throughout all trials. Subjects were asked to provide maximal effort and treat each time trial as a competitive event. For consistency, no verbal encouragement was provided to the subjects during the time trial. The only feedback subjects received during the trials was distance completed during the time trial. To assess the reliability of the data obtained from this performance, a comparable set of male subjects was tested (*N* = 10; 28 ± 8 years, 73 ± 6 kg, 65 ± 9 mL/kg/min). Following a familiarization trial, the coefficient of variation (CV) between two 20 km trials (without nutritional modification) was 1.4% for time, and 2.6% for power output.

### 2.8. Dependent Measurements

*Physiological Responses during Steady-State Exercise*. Oxygen uptake (VO_2_), ventilation (VE), respiratory exchange ratio (RER), heart rate, ratings of perceived exertion (RPE), and blood glucose and lactate were obtained after 5 min of steady-state cycling during EX1 and EX2 (both at 60% W_max_). VO_2_, VE and RER were obtained from expiratory gases using a SensorMedics Spectra metabolic cart (Yorba Linda, CA, USA). Average values were calculated from three minutes of gas collection at each time period, following 2 min of breathing equilibration. Heart rate was obtained using a Polar heart rate monitor (Lake Success, NY, USA). RPE was obtained by having the subject point to their corresponding level of exertion on the Borg (6–20) RPE scale, following a standardized explanation of the scale. An automated YSI 2300 Stat Plus analyzer (Yellow Springs, OH, USA) was utilized to assess glucose and lactate levels from whole blood obtained via finger-stick samples.

*Glucose/Insulin Responses following Beverage Intake*. Thirty minutes after consuming the first recovery beverage, 5 mL of blood was collected using a venous blood draw from an antecubital vein. Blood glucose was assessed as described above. Remaining blood was centrifuged, and serum stored at −80 °C. After being returned to room temperature, serum insulin levels were assessed via a commercially-available ELISA assay (Alpco, Inc.). 

*Muscle Recovery Variables*. Muscle recovery was assessed via muscle soreness ratings, energy/fatigue ratings, serum creatine kinase (CK) levels, and isometric peak torque of the quadriceps (MVC). Soreness ratings were obtained prior to EX1, prior to EX2, and 24 h following EX1. Subjects were asked to subjectively rate the soreness experienced while performing normal daily activities, such as walking, using a 100 mm visual analog scale; with 0 indicating no muscle soreness and 100 indicating impaired movement due to muscle soreness, as described previously [12]. Energy/fatigue ratings were obtained at the same time-points using Part II of the Mental and Physical State and Trait Energy and Fatigue Scales (MPSTEFS [19]). Separate ratings were obtained for Physical Energy, Physical Fatigue, Mental Energy and Mental Fatigue, utilizing “ how do you feel right now” instructions [12]. Each rating represented the combined scores from three visual analog scales of 0–100 mm resulting in potential scores of 0–300 mm for each category. Subjects received instructions regarding how to utilize the muscle soreness and MPSTEFS scales during the familiarization trial.

Voluntary isometric peak force of the right quadriceps was assessed using previously described methodology [12] on five occasions; immediately prior and following EX1 and EX2 (prior to consumption of recovery beverages), and 24 h following EX1. Briefly, subjects performed a maximal leg extension with the right leg on a custom-built muscle function device. The test was conducted in an upright seated position with the leg positioned at approximately 70° of knee flexion. The right leg was used for all subjects to maintain a consistent distance from the axis of rotation of the device. Force measurements were obtained throughout each contraction from a force transducer, and peak force was obtained using custom designed software. Three trials were performed per test, with one minute rest between trials. Peak force (MVC) was recorded as the highest value from the three trials. Participants received practice with this procedure during the familiarization trial. We have previously observed a CV of 6.9% between repeated trials performed in male cyclists tested prior to exercise, with trials separated by approximately one week.

To obtain serum CK, five milliliters of blood was collected using a venous blood draw from an antecubital vein on two occasions: prior to EX1 and 24 h following EX1. Whole blood was spun in a centrifuge at 7000 rpm to separate serum, and stored in a freezer at −80 °C for later analysis. Serum CK was assessed using a Johnson and Johnson Vitro DT 6011 analyzer after samples were returned to room temperature. Prior to analyses, the measurement device was calibrated using a reconstituted lyophilized calibration standard purchased from the manufacturer. All samples were run in duplicate, and mean values were recorded.

*Subsequent Exercise Performance*. Performance following recovery was assessed using cycling time during the simulated 20 km time trial segment of EX2. Average power output and heart rate were also recorded for the trial.

### 2.9. Treatment Beverages

Composition of the recovery beverages (CHO, LCHP, or HCLP) are shown below, and represent the servings provided at each feeding time-point. Beverages were consumed at three time-points: (a) immediately following EX1; (b) 120 min following EX1; and (c) immediately following EX2. 

*Carbohydrate-Only Beverage (CHO).* The CHO beverage contained 300 kcal, and 75 g of carbohydrate (glucose and maltodextrin), per 625 mL serving (12% by volume). The beverage was consumed along with 125 mL of water to provide a total fluid volume of 750 mL.

*Low Carbohydrate*, *High Protein Beverage (LCHP)*. Each 250 mL serving of the LCHP consisted of 290 kcal, 8 g of carbohydrate, 55 g of protein, and 4 g of fat. The protein source was milk-based; including casein and whey protein (as well as providing a small amount of lactose). In order to provide an isovolumetric comparison to other beverages, the LCHP mixture was consumed along with 500 mL of water.

*High Carbohydrate, Low Protein Beverage (HCLP)*. Each 250 mL serving of the HCLP beverage contained 285 kcal, 45 g carbohydrate (glucose and maltodextrin), 25 g milk-based protein (whey and casein, plus leucine), and 0.5 g of fat. Total leucine content in HCLP (3.7 g/serving) was roughly half of that in LCHP (6.3 g/serving). In order to provide an isovolumetric (750 mL) comparison to other beverages, the HCLP mixture was consumed along with 500 mL of water.

Subjects and researchers were blinded to which beverage was consumed during the trials. Beverages were prepared by an assistant who did not participate in the data collection process, and were intended to have similar taste and color. Subjects were not informed regarding the specific contents of the treatments or their potential effects on performance/recovery. However, due to the substantial macronutrient differences between beverages, it is likely that subjects detected different tastes/textures between beverages. Following their final data collection session (*i.e.*, 24 h after their final exercise trial), subjects were asked to rate the carbohydrate and protein content of each of the three beverages used in the study (low, medium or high content). Subjects tended to identify the high carbohydrate content of the CHO beverage (3 low, 2 medium, 7 high), but generally did not note differences in carbohydrate content between LCHP (4, 5, 3) and HCLP (4, 4, 4) beverages. Similarly, subjects tended to note the low protein content in CHO (9, 1, 2), but did not generally identify the higher protein content in the LCHP (2, 7, 3) *versus* HCLP (1, 2, 9). 

### 2.10. Data Analyses

Statistical analyses were conducted using SPSS Version 17.0 (Thomson Learning, Pacific Grove, CA, USA). Power output and total time (from the time-trial portion of EX2), and post-beverage glucose/insulin levels (obtained following EX1) were analyzed using Repeated Measures Analyses of Variance (RMANOVA), with treatment as the within-subject factor. Muscle soreness, MPSTEFS ratings, serum CK and MVC were analyzed using RMANOVA, with treatment and time as within-subject factors. Steady-state responses during exercise (VO_2_, VE, RER, heart rate, RPE, blood glucose, and blood lactate) were analyzed across exercise sessions, using treatment and exercise session (EX1 or EX2) as within-subject factors. These same variables were also assessed within each exercise session (EX1 and EX2) using RMANOVA with treatment as the within-subject factor. A two-tailed alpha level of *p* < 0.05 was utilized for all analyses. Bonferroni adjustments were applied, where appropriate, for multiple comparisons across time-points or treatments.

## 3. Results

*EX1.* The steady-state segment of EX1 (60% W_peak_) was performed at 210 ± 19 W. No significant differences in steady-state responses to exercise were observed between trials (Table 1). High-intensity intervals were conducted at 331 ± 36 W (94 ± 5% W_peak_), 295 ± 33 W (84 ± 5% W_peak_) and 262 ± 31 W (74 ± 5% W_peak_), with recovery intervals performed at 175 ± 16 W (50% W_peak_). Total test duration, number of high-intensity intervals completed, and total energy expenditure (derived from total work completed) during EX1 were not significantly different between CHO (59.2 ± 19.4 min; 13.2 ± 4.8 intervals; 759 ± 298 kcal), HCLP (60.2 ± 19.4 min; 13.4 ± 4.9 intervals; 774 ± 298 kcal) and LCHP (58.6 ± 16.7 min; 13.0 ± 4.2 intervals; 745 ± 264 kcal) trials. 

**Table 1:** Steady State Responses during Exercise (Mean ± SD).

Variable	EX1			EX2		
	CHO	LCHP	HCLP	CHO	LCHP	HCLP
VO_2_ (mL·kg^−1^·min^−1^)	44 ± 5	44 ± 5	44 ± 5	45 ± 4	45 ± 4	44 ± 6
VE (L·min^−1^)	71 ± 6	71 ± 6	71 ± 8	70 ± 7	70 ± 6	68 ± 4
RER ^#^	0.93 ± 0.04	0.93 ± 0.03	0.92 ± 0.03	0.91 ± 0.03	0.89 ± 0.03	0.91 ± 0.03
HR(bt·min^−1^) ^#^	152 ± 10	149 ± 10	148 ± 11	153 ± 10	153 ± 8	150 ± 9
RPE ^#^	12.8 ± 1.3	12.5 ± 1.6	12.8 ± 1.4	13.4 ± 1.1	12.9 ± 1.7	13.1 ± 1.5
Glucose (mg·dL^−1^) ^@^	65.8 ± 9.9	66.0 ± 8.7	62.2 ± 6.9	58.8 ± 9.7 *^,^^	66.9 ± 8.6	67.4 ± 6.3
Lactate (mmol·L^−1^) ^#^	2.6 ± 1.1	2.7 ± 1.0	2.7 ± 1.2	1.9 ± 0.8	1.5 ± 0.7	1.9 ± 0.7

Note: EX1 conducted prior to ingestion of treatment beverages. Beverages: CHO = carbohydrate; LCHP = low carbohydrate, high protein; HCLP = high carbohydrate, low protein; * = different from LCHP (within EX2), *p* < 0.05; ^ = different from HCLP (within EX2), *p* < 0.05; ^#^ = main-effect for exercise bout (EX1-EX2), *p* < 0.05; ^@^ = treatment × exercise bout interaction, *p* < 0.05.

*Glucose/Insulin during Recovery.* Glucose levels obtained 30 min post-feeding were significantly different (*p* < 0.05) between all treatments (CHO = 110.6 ± 17.5 mg·dL^−1^; HCLP = 88.0 ± 16.9 mg·dL^−1^; LCHP = 71.6 ± 8.9 mg·dL^−1^; *p* < 0.05). Insulin levels were significantly lower (*p* < 0.05) for LCHP (31 ± 10 µU·mL^−1^) than both of the other treatments, with no differences between CHO (133 ± 63 µU·mL^−1^) and HCLP (113 ± 31 µU·mL^−1^). Due to technical issues with the initial analysis, inadequate sample volumes were available to analyze insulin from all trials. Thus, the insulin values reported are from 4 subjects with samples available for all three trials. However, the results of the RMANOVA (LCHP < HPLC = CHO) were confirmed in individual comparisons between treatments (*i.e.*, *t*-tests between the individual treatments, which increased sample sizes to 5–7 per comparison). 

*EX2.* Physiological data obtained from the steady-state segment of EX2 are shown in Table 1. Blood glucose levels were significantly lower during the CHO trial than both the HCLP and LCHP trials. No other significant differences were observed between treatments. Data were also compared between EX1 and EX2. VO_2_ and VE were not different between the two exercise sessions, and exhibited no treatment × exercise session interactions. HR and RPE were significantly higher during EX2, but were not significantly influenced by treatment. RER and lactate were significantly lower during EX2. No treatment × exercise session interactions were observed for these variables. No changes in mean blood glucose were noted between EX1 and EX2, but a significant treatment × exercise session interaction was present. 

**Figure 2:**
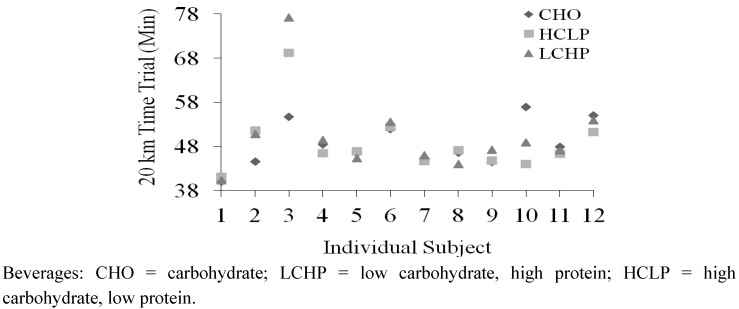
Individual Performance Times during EX2 Time-Trial.

Exercise time, power output and heart rate for the time-trial segment of EX2 are shown in Table 2. No significant differences were observed between any treatments for these variables. Individual performances across the trials are shown in Figure 2. 

**Table 2:** Performance Variables during Subsequent Exercise Time-trial (Mean ± SD).

Variable	CHO	LCHP	HCLP
Total time for 20 km (min)	48.5 ± 5.1	50.3 ± 9.3	48.8 ± 7.2
Average power for 20 km (W)	204 ± 34	198 ± 43	205 ± 37
Average HR for 20 km (bt·min^−1^)	156 ± 10	152 ± 12	156 ± 6

Beverages: CHO = carbohydrate; LCHP = low carbohydrate, high protein; HCLP = high; carbohydrate, low protein; No differences between treatments (*p* > 0.05).

**Figure 3:**
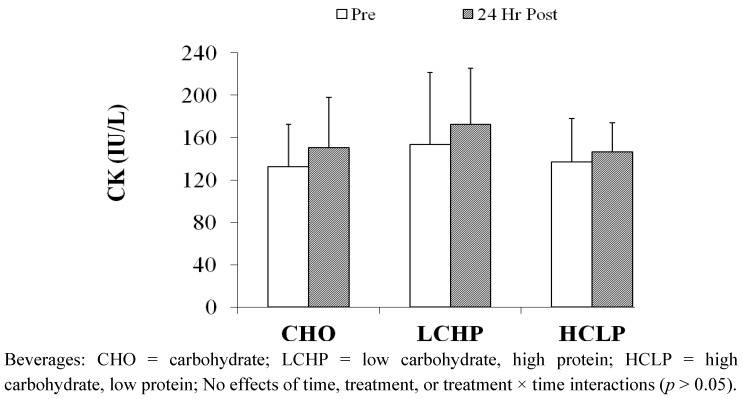
Influence of Exercise and Recovery Beverages on Serum CK Levels (Mean ± SD).

**Figure 4:**
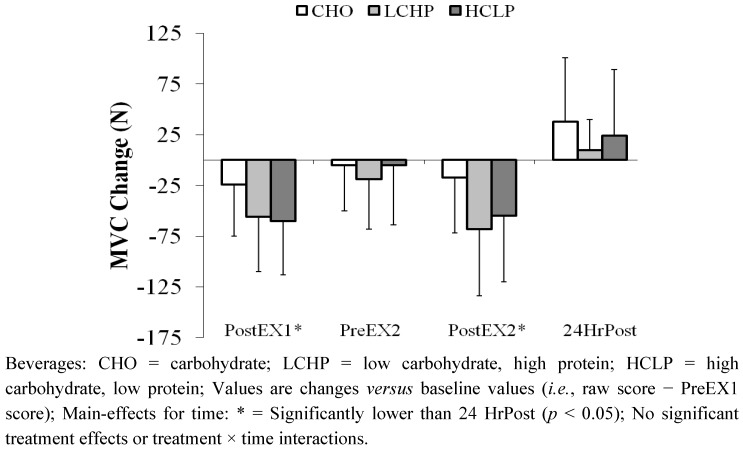
Influence of Exercise and Recovery Beverages on MVC (Mean ± SD).

*Muscle Recovery Variables.* CK responses are reported in Figure 3. No significant effects were observed for time, treatment or treatment × time interaction. Isometric MVC values (reported as change scores: raw score − PreEX1 score) are shown in Figure 4. A significant main effect was observed for time, with Post-EX1 and Post-EX2 values (independent of treatment) significantly lower than those obtained 24 h post-exercise. No significant treatment or treatment × time interactions were observed for MVC. 

Subjective ratings of muscle soreness and energy/fatigue (MPSTEFS) are shown in Table 3. Each of these ratings was significantly altered between pre-EX1 and pre-EX2 (independent of treatment). MPSTEFS ratings were not significantly different between pre-EX1 and 24 h post-exercise. However, muscle soreness levels remained significantly elevated 24 h post-exercise *versus* pre-EX. No significant treatment or treatment × time effects were observed for any of the ratings.

**Table 3:** Ratings of Physical and Mental Energy/Fatigue and Muscle Soreness (Mean ± SD).

Variable	CHO			LCHP			HCLP		
	PreEX1	PreEX2	24Post	PreEX1	PreEX2	24Post	PreEX1	PreEX2	24Post
Physical Energy ^@^^,#^	200 ± 63	160 ± 61	200 ± 64	193 ± 46	161 ± 56	193 ± 63	194 ± 64	160 ± 49	181 ± 69
Physical Fatigue ^@^^,#^	79 ± 59	148 ± 66	88 ± 61	88 ± 47	143 ± 57	91 ± 54	83 ± 59	138 ± 53	106 ± 63
Mental Energy ^@^^,#^	195 ± 70	175 ± 62	201 ± 67	201 ± 54	169 ± 60	194 ± 66	198 ± 54	172 ± 59	191 ± 66
Mental Fatigue ^@^^,#^	94 ± 67	117 ± 61	89 ± 70	88 ± 53	117 ± 62	90 ± 55	81 ± 52	117 ± 61	89 ± 59
Muscle Soreness ^@^^,#^^,&^	10 ± 11	36 ± 18	25 ± 23	13 ± 10	33 ± 15	19 ± 12	16 ± 17	39 ± 19	25 ± 17

Beverages: CHO = carbohydrate; LCHP = low carbohydrate, high protein; HCLP = high carbohydrate, low protein; All variables recorded in mm, from VAS. Significant main-effects for time: ^@^ = PreEX1 different than PreEX2 (*p* < 0.05); ^#^ = PreEX2 different than 24 Post (*p* < 0.05); ^&^ = PreEX1 different than 24 Post (*p* < 0.05).

## 4. Discussion

The purpose of the present study was to determine if beverages with similar caloric content, but substantial variations in carbohydrate and protein content produced different effects on post-exercise recovery. Specifically, we assessed these effects during short-term recovery following an exercise session of ~60 min of high-intensity cycling intervals. Blood glucose levels obtained 30 min after the first ingestion of the recovery beverages (post-exercise) were significantly different between treatments, in proportion to the amount of carbohydrate in each beverage (CHO > HCLP > LCHP). In addition, serum insulin levels at this same time-point were higher in the CHO and HCLP trials compared to the LCHP trial. The similar insulin response between HCLP and CHO trials is consistent with prior studies of similarly proportioned CHO + Pro beverages, which have generally reported equal or greater hyperinsulinemic responses following CHO + Pro ingestion; as recently reviewed by Betts and Williams [5]. The lower insulin response with the LCHP beverage was likely related to its low carbohydrate content (8 g per feeding *versus* 45 and 75 g for HCLP and CHO, respectively) and could have potentially influenced glycogen replenishment rates in this trial, though glycogen levels were not assessed. During EX2, steady-state values for HR and RPE were increased, by a small degree, compared to EX1 (independent of treatment). In addition, RER and blood lactate levels were lower during EX2, suggesting a decreased reliance on carbohydrate during the steady-state segment of the subsequent exercise trial. The mean values for these variables were both slightly lower in the LCHP trial compared to the CHO and HCLP trials (which were identical). Neither effect was statistically significant between treatments, but these findings suggest a possible tendency towards reduced carbohydrate availability in some individuals in the LCHP trial. When combined with the lower insulin response in the LCHP trial, there is evidence that this beverage may have been less effective at promoting recovery in some individuals compared to HCLP and CHO. However, no significant differences in subsequent exercise performance were observed between the CHO (48.5 ± 1.5 min), HCLP (48.8 ± 2.1 min) and LCHP (50.3 ± 2.7 min) recovery beverages. The mean value for LCHP could represent a “practically significant” impairment in performance (*i.e.*, 1.5–1.8 min) if consistently observed across subjects. However, statistical analyses did not support evidence for systematic differences between treatments (*p* = 0.339 and 1.00 for caparisons of LCHP *versus* HCLP and CHO, respectively). Thus, we conclude that the specific macronutrient composition of the recovery beverages (in the absence of substantial caloric differences between treatments) had little effect on short-term recovery under the conditions tested in this study. 

Over the past decade, numerous investigators have examined the effects of CHO + Pro co-ingestion on subsequent exercise performance. Several studies have observed enhanced performance with CHO + Pro intake *versus* carbohydrate-only treatments (*i.e.*, [4,7,10,13,20,21]). However, other studies have reported no differences in subsequent exercise performance compared to CHO intake following exercise (*i.e.*, [2,3,6,11,14,22,23]). The findings of the present study are generally consistent with this latter group of investigations. Of particular relevance are findings from Betts and colleagues [23]. These investigators observed that post-exercise CHO + Pro ingestion enhanced performance in a subsequent bout of exercise when compared to a CHO beverage with equal carbohydrate content (but fewer calories); but not in comparison to an isocaloric CHO beverage. Some prior studies [4,7,10,13] have reported that CHO + Pro ingestion enhances subsequent exercise performance *versus* isocaloric CHO treatments. However, the present data suggest that the specific proportions of carbohydrate and protein may elicit only subtle influences on subsequent performance when comparing beverages that are similar in caloric content, at least under the present study conditions. 

The potential mechanisms explaining how CHO + Pro may influence recovery are not clearly defined. However, there is evidence that CHO + Pro ingestion following endurance exercise may have positive effects on glycogen resynthesis [5,24], protein turnover [25,26], rehydration [27], and markers of muscle disruption [2,28]; which may individually or collectively result in augmented performance in subsequent exercise. Thus, it is possible that the lack of consistent findings across different studies may be related to the varied effects of differing exercise/nutrition protocols on these aforementioned factors. One aim of the present study was to assess the effects of the different beverages on markers of muscle disruption, as a potential mechanism for changes in performance. We assessed a variety of post-exercise recovery measurements, including serum CK levels, muscle function (MVC), muscle soreness and energy/fatigue ratings; and all were similar between treatments. These findings support the interpretation that the CHO, LCHP, and HCLP beverages provided similar effects on post-exercise recovery. Prior studies investigating the effects of CHO + Pro ingestion on these variables have been mixed, with numerous studies reporting positive effects of CHO + Pro, and others reporting no difference *versus* CHO treatments (see recent review from Saunders [29]). Notably, the present findings differ from previous studies from our own laboratory, which have reported that CHO + Pro improved markers of recovery after heavy cycling exercise *versus* isocaloric CHO beverages [6,8]. The present study utilized a shorter duration exercise protocol than our prior studies (~60 min *versus* >95 min; albeit at a higher intensity), which may have influenced study outcomes. However, other investigators have reported positive effects of CHO + Pro beverages on recovery following cycling bouts of similar exercise intensity and/or duration [4,15,16], even when compared to isocaloric CHO beverages [4]. In addition, Lunn and colleagues [30] recently reported that subsequent performance was improved when CHO + Pro (chocolate milk) was consumed during recovery from a 45 min run at 65% VO_2max_. Thus, there is sufficient evidence that post-exercise nutritional strategies have the potential to promote recovery following similar (or even less demanding) bouts of exercise than investigated here. 

Betts and associates [9] speculated that CHO + Pro supplementation may only be effective at attenuating less severe degrees of exercise-induced muscle damage; as they observed that most (though not all) studies reporting positive influences of CHO + Pro on recovery have reported moderate changes in post-exercise serum/plasma CK levels (250–600 U/L). Saunders [29] also noted that negligible levels of impairment following initial exercise may prevent any possible treatment effects from being observed, as the small effect-sizes would limit statistical sensitivity. Thus, it is possible that the influences of CHO + Pro ingestion may not be detected in studies with very small or large changes in muscle disruption following the initial exercise session. This observation may explain the absence of significant treatment effects in the present study. The observed changes in recovery variables indicate a relatively small degree of muscular impairment in all treatment conditions. For example, post-exercise MVC values were decreased immediately following each exercise session (Figure 4), but values prior to subsequent exercise were not substantially different from baseline levels. Serum CK levels were not significantly elevated 24-h after exercise (Figure 3). MPSTEFS ratings were slightly impaired prior to subsequent exercise, but had returned to baseline levels within 24-h (Table 3). The only variable that remained altered at 24-h post-exercise was muscle soreness (Table 3), and this change was substantially smaller than in prior studies using similar soreness scales (*i.e*., [18]). Collectively, these data indicate that the subjects recovered effectively, and were minimally “impaired” under all treatment conditions. The absence of substantial changes in 24-h recovery measurements differs from most prior investigations. For example, previous studies from our laboratory have reported significant increases in post-exercise CK levels in cyclists receiving CHO intake during and/or following exercise [6,8,20,31,32]. 

The possibility that the initial exercise session was not demanding enough for subjects to benefit from any recovery beverage (or nutrients) during the recovery period cannot be entirely refuted without a non-caloric placebo beverage for comparison. However, as previously discussed, numerous studies have reported significant differences in subsequent exercise with nutritional intervention following similarly demanding bouts of exercise [4,15,16,30]. The 1 h exercise session (EX1) included approximately 26 min of work at 262–331 W. This was freely noted by many subjects to be a very difficult workout, and the high-intensity intervals led to volitional fatigue in all participants. Regardless, the current findings provide additional insight regarding the conditions in which CHO + Pro may (or may not) be useful for recovery. Although some studies (including those from our own laboratory) have concluded that CHO + Pro enhances recovery *versus* carbohydrate alone, the present study revealed no significant influences on recovery and subsequent performance following ~1 h of heavy exercise in well-trained cyclists. Thus, the putative benefits of CHO + Pro appear to be condition-specific. The absence of treatment effects on recovery in the present study could have been influenced by the somewhat short exercise duration (discussed above), or relatively high training/fitness status in this cohort of subjects (*i.e.*, mean VO_2peak_ values were higher than any of our prior studies [6,8,18,20,32]); which could be related to enhanced recovery capabilities. In addition, post-exercise recovery responses vary considerably among subjects within any given study. Thus it is possible that our sample included a group of subjects who recovered disproportionately well following exercise, regardless of macronutrient availability. These issues require subsequent investigation in order to ascertain the specific conditions in which CHO + Pro beverages may promote enhanced recovery in athletes. Another potential limitation of the present study design was the use of commercial nutrition products, which prevented the matching of all ingredients in the beverages. Although the variations between beverages were likely trivial in comparison to their macronutrient differences (*i.e.*, small differences in calories, slight variations in protein and carbohydrate sources), any treatment effects cannot be attributed entirely to differences in protein per second. In addition, the validity of some markers of muscle recovery (such as CK levels and muscle soreness ratings) have been questioned, as they may not be strongly correlated with direct markers of muscle damage or muscle function [33]. The repeated-bout effect may also influence the magnitude of changes in muscle recovery [34], increasing error variance in studies of post-exercise recovery. While the potential influences of these factors on study outcomes cannot be discounted, the use of cycling exercise (which has minimal eccentric muscle contractions), randomly counterbalanced exercise trials, well-trained subjects, and a familiarization trial in the present protocol minimized the potential influence of any repeated-bout effect. In addition, the observed changes in MVC (considered a valid method of quantifying muscle injury) [33] throughout the trials were consistent with changes in other measures of recovery, suggesting that the lack of treatment effects was not merely the result of poor measurement sensitivity. 

## 5. Conclusions

In summary, each of the beverages (CHO, HCLP, LCHP) provided similar effects on recovery following heavy aerobic exercise, despite variations in the carbohydrate/protein compositions of the beverages. Thus, following heavy aerobic exercise of approximately 1 h duration, short-term exercise recovery of well-conditioned male cyclists does not appear to be significantly influenced by the macronutrient content of recovery beverages, provided that the caloric content of the beverages is similar (*i.e.*, 285–300 kcal in each serving). These findings may be limited to well-trained endurance cyclists, as it is possible that populations that incur larger disruptions in muscle recovery may obtain different results from the treatment beverages. Therefore it is recommended that future investigations examine the effects of CHO + Pro beverages under differing exercise conditions, and with participants of varying training status. 
